# Nutrition knowledge among university students in the UK: a cross-sectional study

**DOI:** 10.1017/S1368980021004754

**Published:** 2022-10

**Authors:** Katerina Belogianni, Ann Ooms, Anastasia Lykou, Hannah Jayne Moir

**Affiliations:** 1 Faculty of Health, Social Care and Education, Kingston University & St George’s, University of London, Kingston upon Thames, UK; 2 Department of Nutritional Sciences, King’s College London, 150 Stamford Street FWB, Room 4.103, London SE1 9NH, UK; 3 Department of Education, University of Nicosia, Nicosia, Cyprus; 4 Faculty of Science, Engineering and Computing, Kingston University London, Kingston upon Thames, UK

**Keywords:** University students, Universities, Knowledge, Diet, Health, Young adults

## Abstract

**Objective:**

To investigate nutrition knowledge (NK) in university students, potential factors affecting knowledge and predictors of good NK.

**Design:**

A cross-sectional study was conducted in 2017–2018. The revised General Nutrition Knowledge Questionnaire was administered online to assess overall NK and subsections of knowledge (dietary recommendations, nutrient sources of foods, healthy food choices and diet–disease relationships). The Kruskal–Wallis test was used to compare overall NK scores according to sex, age, ethnicity, field of study, studying status, living arrangement, being on a special diet and perceived health. Logistic regression was performed to identify which of these factors were associated with a good level of NK (defined as having an overall NK score above the median score of the sample population).

**Setting:**

Two London-based universities.

**Participants:**

One hundred and ninety students from various academic disciplines.

**Results:**

The highest NK scores were found in the healthy food choices (10 out of 13 points) and the lowest in the nutrient sources of foods section (25 out of 36 points). Overall NK score was 64 out of 88 points, with 46·8 % students reaching a good level of knowledge. Knowledge scores significantly differed according to age, field of study, ethnicity and perceived health. Having good NK was positively associated with age (OR = 1·05, (95 % CI 1·00, 1·1), *P* < 0·05), White ethnicity (OR = 3·27, (95 % CI 1·68, 6·35), *P* < 0·001) and health rating as very good or excellent (OR = 4·71, (95 % CI 1·95, 11·4), *P* < 0·05).

**Conclusions:**

Future health-promoting interventions should focus on increasing knowledge of specific nutrition areas and consider the personal and academic factors affecting NK in university students.

The transition to university can be a turbulent period of a young persons life, characterised by increased independency, socialising, self-regulation and self-organisation^(1)^. Qualitative research among university students have shown that dietary habits are driven by a cluster of personal, societal, environmental and academic factors^(2)^. Among societal and environmental parameters are the influence of peers and the availability and affordability of foods. Nutrition knowledge (NK) and perceived health benefits of food, together with other individual factors (e.g. cooking skills), are also factors affecting dietary behaviour according to students^(2)^. NK in university students has been found to positively associate with an increased intake of fruit, dairy, protein and wholegrain foods^(3)^ or other dietary behaviours (i.e. reading food labels)^(4)^. Findings from existing cross-sectional studies in university students suggest an inadequate knowledge of various nutritional topics. In particular, students failed to correctly answer more than 50 % of the questions in relation to fruit and vegetables^(5)^, milk or their alternatives and fermented dairy products^(5)^, vitamin D^(6,7)^, food labels^(8)^ and the impact of diet on chronic diseases^(4,9,10)^.

The General Nutrition Knowledge Questionnaire (GNKQ) developed by Parmenter & Wardle (1999)^(11)^ in the UK is a validated tool to assess NK in adults and has also been used previously in studies with university students^(4,9,12,13)^. Studies among university students, which assessed knowledge using the GNKQ tool, found that the mean scores of correct answers ranged from 51 % to 67 %, suggesting a moderate level of overall knowledge^(4,9,12,13)^. Kliemann *et al.*^(14)^ published in 2016 a revised version of the GNKQ including updated evidence-based information on nutritional facts and dietary recommendations. The revised version included questions on dietary recommendations according to the UK Eatwell Guide published in 2016^(15)^, hidden sources of salt and added sugars, food labelling and cooking methods, as well as glycaemic index of foods, body shape and optimal practices to maintain a healthy body weight.

Academic discipline, sex, age and socio-economic parameters are factors that might affect the level of NK. Having received nutritional education^(16)^ or studying a health-related course^(17)^ has also been associated with increased knowledge in some students. An increased level of knowledge has also been reported in older students when compared to their younger counterparts^(18,19)^ as well as female students^(20,21)^. Additional studies found that high socio-economic status^(10)^, healthy BMI^(22)^, non-Hispanic White race^(20)^ and living alone^(12)^ were positively associated with greater NK in university students. These studies were not undertaken in Europe, which highlights the gap in the European literature on this topic. In the UK, however, a similar study among university students assessed NK as a predictor of diet quality, using the initial version of the GNKQ^(4)^. The study showed that socio-demographic characteristics had an impact on NK, while NK was a significant predictor of diet quality.

By increasing the knowledge of students with regard to nutrition and healthy eating, students are given the opportunity to personalise this knowledge to improve their diet quality. Considering that students from non-health academic disciplines (e.g. Political Sciences, Mathematics) might never have the chance to receive evidence-based nutritional information via their courses, it is important to include nutrition information in any health-promoting strategy. The objectives of this study were to explore the level of NK in a sample of university students in the UK from various academic disciplines, investigate potential factors affecting knowledge and explore predictors of good NK. Understanding the current level of students’ knowledge contributes towards the design of targeted and more successful interventions within university settings.

## Methods

### Study population and design

This cross-sectional study took place in two London-based UK Higher Education Institutions which provide both health (e.g. Medicine, Nursing, Midwifery, Physiotherapy) and non-health courses (e.g. Engineering, Art and Design, Business), giving the opportunity to recruit students across different academic disciplines. The study was approved by the two universities’ Ethics Committee. Eligible participants were students from all ages (above 18 years old), independent of their mode of attendance (part-time or full-time) or studying status (undergraduate or postgraduate) with no further exclusion criteria applied. The link to the online survey was circulated via both universities’ electronic mail systems and the online survey was open during one academic year (2017–2018). The survey was anonymous, participation was voluntary and no survey questions were mandatory. Informed consent was obtained by clicking to ‘agree’ with consent statements prior to entering the survey. Power sample size was calculated based on Kliemann *et al*.^(14)^, which found that the standard deviation of the NK score of non-dietetic students is 9·2. Assuming a mean score of 65 in our sample (scores range from 0 to 88) and a sd of 9·2, a sample size of 180 participants was considered sufficient.

### Nutrition knowledge

NK was assessed using the revised General Nutrition Knowledge Questionnaire (GNKQ-R)^(14)^. The GNKQ-R assesses the following four sections of NK: (1) dietary recommendations (eighteen items); (2) nutrient sources of foods (thirty-six items); (3) healthy food choices (thirteen items); and (4) diet–disease relationships and weight management (twenty-one items) and overall knowledge (sum of all sections, totalling eighty-eight items). Examples of questions in the first section included whether experts recommend eating more or less foods from various groups (e.g. fruits, vegetables, wholegrains, oily fish and fats) as well as the recommended servings according to the UK Eatwell Guide. Examples of questions in the second section included whether specific foods (breakfast cereals, ketchup, cheese, etc.) are high or low in added sugars, salt, fibre, protein or specific type of fats. In the third section, participants were asked to choose the healthiest option from a list of meals, desserts or foods cooked in different ways. Examples of questions in the fourth section included whether the intake of specific foods and nutrients such as red meat, sugar and fibre increases or decreases the risk of diseases such as cancer and type 2 diabetes. This section also included questions about good practices to maintain a healthy body weight, such as reading food labels and avoid grazing throughout the day. Each question had only one option and the correct answer was given one point (otherwise null). The GNKQ-R has high internal consistency (Cronbach’s *α* > 0·7) and external reliability (intraclass correlation coefficient >0·7) in all sections^(14)^. Before administration, a pilot study was undertaken to estimate the feasibility of the survey.

Demographic- and academic-related questions were included at the end of the GKNQ-R survey. Students were asked about their sex (i.e. male and female), age (years), university enrolled, Faculty enrolled (i.e. Arts and Social Sciences; Health, Social Care and Education; Business and Law; Art, Design and Architecture; Science, Engineering and Computing and Medicine; Biomedical Sciences), studying status (i.e. undergraduate and postgraduate), having any nutrition qualification (i.e. yes and no), current living arrangement (i.e. living with parents/carer/family, sharing a house or flat, living in a student accommodation and living alone in a house or flat), ethnicity (i.e. White, Black, Asian, other or mixed background), perceived health (e.g. poor, fair, very good and excellent), being on a special diet (e.g. yes or no with further clarification on the type of the diet), body weight, stature and whether being a smoker (i.e. yes and no).

### Data analysis

Initially, 301 participants entered the study, of which 249 participants provided consent and 190 completed at least 90 % of the questionnaire and were included in the final analysis. Multiple imputation was performed to account for the missing values, under the missing at random assumption. Missing values of scores, along with students’ demographic and academic characteristics, were replaced with imputed values (five imputed values were selected for each missing cell) and the analysis was performed for all five datasets. Statistical analysis was performed using the statistical program R and the package Amelia II^(23)^ (for missing values imputation) and the Statistical Package for the Social Sciences (IBM SPSS Statistics for Windows, Version 24.0). The statistical significance level was set at *P* ≤ 0·05.

The Shapiro–Wilk test as well as Q-Q (quantile-quantile) probability and cumulative frequency plots were used to determine the normality of data distribution. The null hypothesis of the test was rejected for GKNQ-R (*P* < 0·001); therefore, non-parametric tests were used in the data analyses. Descriptive characteristics of the participants are presented as means and standard deviations or as absolute and relevant frequencies. Descriptive statistical analyses were performed to calculate the median scores and interquartile ranges of each knowledge section and overall NK.

The Kruskal–Wallis test was used to compare the median values of overall GNKQ-R scores in the various groups of students. The categorical variable ‘field of study’ was created based on the Faculty of study to group students into healthcare and non-healthcare field of study. The healthcare field of study included students from the Faculties of Health, Social Care and Education, Medicine and Biomedical Sciences and those from other Faculties holding a nutrition qualification. Students from the remaining Faculties and with no nutrition qualification were included in the non-healthcare field of study. The median score of overall NK of the sample population was used as a cut-off point to indicate the level of NK, as suggested in similar studies^(18,24)^. Students with scores equal to or above than this value were categorised as having ‘good’ and those with lower values were categorised as having ‘poor’ NK. Chi-square (*χ*^2^) tests were used to examine the level of NK (‘poor’/‘good’) according to sex, age, ethnicity, field of study, studying status, living arrangement, being on a special diet and perceived health.

Binary logistic regression analysis was performed to identify significant predictors of good NK (dependent variable). A stepwise forward variable selection was used to identify all independent variables with a significant bivariate crude association with the dependent variable. Prior nutrition qualification was excluded from the analysis as it interacted with the ‘field of study’ variable, while sex and BMI variables were included, despite no significant association being found, as evidence suggests they are predictors of NK ^(4,21)^. The analysis was performed for all five imputed datasets, returning similar results. For simplicity reasons, the findings of a single dataset are presented.

## Results

The characteristics of the study population are presented in Table 1. The majority of students were female (68·9 %), of White ethnicity (59·5 %), undergraduate (78·4 %) and younger than 25 years old (62·6 %). The final sample had a mixed population, with 41·1 % of students enrolled in a healthcare course and 58·9 % enrolled in a non-healthcare course. About one-third of students (33 %) were living with their family or sharing a house and one-quarter of students (24·7 %) were living in student accommodation. When asked to rate their health, 27·4 % perceived their health as ‘very good’ or ‘excellent’. The majority of students (64·2 %) had a normal BMI with a mean value of 24·6 ± 5·7 kg/m^2^ and about one-third (35·8 %) belonged to the overweight/obese BMI category (i.e. BMI ≥ 25 kg/m^2^). Finally, very few students (14·2 %) reported being on a special diet (e.g. vegetarian and vegan).

**Table 1:** Description of demographic and academic characteristics of the sample population (*n* 190)

Variable	Number of participants (*n*)	Percentage of cohort (%)
Age (years)		
≤ 25	119	62·6
>25	71	37·4
Mean	25·7	
sd	8·1	
Sex		
Female	131	68·9
Male	59	31·1
Ethnicity		
White*	113	59·5
Black†, Asian‡, Mixed/Other§	77	40·5
Studying status		
Undergraduate	149	78·4
Postgraduate	41	21·6
Field of study		
Healthcare||	78	41·1
Non-healthcare	112	58·9
Living arrangement		
With parent(s)/carer/family	63	33·2
Sharing a house or flat	64	33·7
Student accommodation	47	24·7
Alone (in house/flat)	16	8·4
Perceived health		
Poor/fair	58	30·5
Good	80	42·1
Very Good/excellent	52	27·4
Being on a special diet		
No	163	85·8
Yes	27	14·2
Being a smoker		
No	166	87·4
Yes	24	12·6
BMI (kg/m^2^)		
Underweight/normal weight (<25·0)	122	64·2
Overweight (≥25)	68	35·8
Mean	24·6	Min = 16, max = 53
sd	5·7	

* White British, White Irish or other White ethnic background.

† Black British, Black Caribbean, Black African or other Black ethnic background.

‡ Indian, Pakistani, Bangladeshi, Chinese or other Asian ethnic background.

§ White and Black Caribbean, White and Black African, White and Asian or other mixed ethnic background.

|| The healthcare field included students from the Faculty of Health, Social Care and Education and the Medicine/Biomedical Sciences and those from other Faculties with a nutrition qualification. All other students were included in the non-healthcare field.

Students had an overall NK median score of 64 out of 88 points (72·7 %) (Table 2). With regard to the subsections of knowledge, students had a median score of 14 out of 18 points on the section of dietary recommendations; a median score of 25 out of 36 points on the section of nutrient sources of foods; a median score of 10 out of 13 points on the section of healthy food choices and a median score of 15 out of 21 points on the section of diet–disease relationships and weight management. Students’ responses to individual questions were further investigated to gain a better understanding of their knowledge within each section (data not shown). With regard to dietary recommendations, about half of the students were not aware of the recommendations of increasing wholegrain intake, reducing alcohol intake to one drink both for men and women, two glasses of fruit juice count only as one serving of fruit and starchy foods should make up a third and not a quarter of our diet. With regard to food groups and the nutrients they contain, less than half of the participants identified that breakfast cereals and bread are hidden sources of salt and about half of them were not aware that regular pasta has a low fibre content opposed to plantains which have a high fibre content. When asked about the type of fat contained in various foods, only one in five students reported that sunflower oil is rich in polyunsaturated fat, one in four reported that olive oil is rich in monosaturated fat and one in three reported that eggs are rich in cholesterol, with many students choosing ‘not sure’ as an option to these questions. In the section of healthy food choices, students performed well in general and managed to select the healthiest option when asked about different types of meals, drinks and desserts. However, their knowledge was not as strong when asked which cooking method, that is, sauteing, grilling or baking, requires fat to be added, with only one-third of participants choosing sauteing as the correct answer. In the last section of diet and disease relationships and weight management, about half of the students reported correctly that eating less red meat helps prevent cancer and that a high protein diet does not help to maintain a healthy weight.

**Table 2:** Nutrition knowledge of the sample population (*n* 190)

Nutrition knowledge	Maximum knowledge score	Median score	IQR
Dietary recommendations	18	13·5	3
Nutrient sources of foods	36	25·0	6
Healthy food choices	13	10·0	3
Diet–disease relationships and weight management	21	15·0	4
Overall nutrition knowledge	88	64·0	12

	Level*	*n*	%
	Good	89	46·8
	Poor	101	53·2

GNKQ-R, General Nutrition Knowledge Questionnaire-Revised.

* Good nutrition knowledge is defined as having an overall median GNKQ-R score ≥ 64 points and poor knowledge as having a score <64 points.

The median scores of overall NK among the different groups of students as well as the number of students with ‘poor’ or ‘good’ levels of NK for each group are presented in Table 3. In particular, the median scores of knowledge were higher for students in the healthcare field of study compared to students in the non-healthcare field of study (66·0 *v*. 62·0, *P* < 0·05). However, the number of students with a ‘good’ or ‘poor’ level of NK did not differ significantly within each group (*P* = 0·106). Students of White ethnicity also had higher median scores of NK than students of Black, Asian or other/Mixed ethnicity (66·0 *v*. 61·0, *P* < 0·001), with 70·1 % of students of the Black, Asian and other or Mixed ethnic groups demonstrating ‘poor’ level of NK and 29·9 % demonstrating ‘good’ level of NK (*P* < 0·001). Similarly, students with a ‘good’/‘excellent’ perceived health had higher median scores of NK compared to students who perceived their health as ‘good’ or ‘poor’/‘fair’ (68·0 *v*. 63·5 *v*. 61·0, *P* < 0·05), with 70·7 % of students in the ‘poor’/‘fair’ category demonstrating ‘poor’ level of NK and 29·3 % demonstrating ‘good’ level of NK (*P* < 0·001). A marginally significant trend was found for age, with students aged 25 years or above 25 years having higher median NK scores compared to their younger counterparts (*P* = 0·049); however, no significant differences were found for the level of knowledge within each group. No significant differences in median scores or level of knowledge were found according to sex (*P* = 0·145), BMI (*P* = 0·846), studying status (*P* = 0·460), living arrangements (*P* = 0·229) and being on a special diet (*P* = 0·134).

**Table 3:** Number of students with poor or good level of nutrition knowledge and the median scores of overall nutrition knowledge by socio-demographic and other categorical variables in the student population (*n* 190)

Variable	Overall nutrition knowledge	Poor nutrition knowledge		Good nutrition knowledge*	
	Median scores	*n* within group variable	%	*n* within group variable	%
Age category					
≤25 years	62·0	68	57·1	51	42·9
>25 years	66·0	33	46·5	38	53·5
	*P* = 0·049	*X*^2^ = 2·03, *P* = 0·154			
Field of study					
Healthcare	66·0	36	46·2	42	53·8
Non-healthcare	62·0	65	58·0	47	42·0
	*P* = 0·042	*X*^2^ = 2·61, *P* = 0·106			
Ethnicity					
White	66·0	47	41·6	66	58·4
Black, Asian, Mixed/Other	61·0	54	70·1	23	29·9
	*P* = 0·000	*X*^2^ = 15·0, *P* = 0·000			
Perceived health					
Poor/fair	61·0	41	70·7	17	29·3
Good	63·5	43	53·8	37	46·3
Very Good/excellent	68·0	17	32·7	35	67·3
	*P* = 0·001	*X*^2^ = 16·0, *P* = 0·000			

GNKQ-R, General Nutrition Knowledge Questionnaire-Revised.

* Good knowledge is defined as having an overall median GNKQ-R score ≥ 64 points.

The logistic regression analysis showed that age, perceived health and ethnicity were significant predictors of good NK (Table 4). In particular, students who rated their health as ‘very good’ or ‘excellent’ were 4·7 times more likely to have ‘good’ NK compared to students who rated their health as ‘poor’ or ‘fair’ (OR = 4·71, (95 % CI 1·95, 11·37), *P* < 0·05). Similarly, those of White ethnicity were three times more likely to have ‘good’ NK compared to students of ethnicity other than White (OR = 3·27, (95 % CI 1·68, 6·35), *P* < 0·001). An association was also found between age and knowledge, as a 1-year increment in age could increase the level of ‘good’ NK by 5 % (OR = 1·05, (95 % CI 1·00, 1·1), *P* < 0·05). No significant association was found between sex (*P* = 0·672), BMI (*P* = 0·733) and field of study (*P* = 0·609) and ‘good’ NK. The overall model predicted 17·1 % of the dependent variable (Model Summary *R*^2^ = 0·171, *P* < 0·001).

**Table 4:** Predictors of good nutrition knowledge among university students (*n* 190)

Dependent variable	Predictors	OR	95 % CI		*P*-value
			Lower	Upper	
Good nutrition knowledge	Age (years)	1·05	1·00	1·1	0·041
	Perceived health				
	Poor/fair (RG)				
	Good	1·79	0·82	3·91	0·144
	Very good/excellent	4·71	1·95	11·4	0·001
	Ethnicity				
	Black/Asian/Mixed/Other (RG)				
	White	3·27	1·68	6·35	0·000

RG, reference group.

The full model included age, BMI, perceived health, field of study, ethnicity and sex.

## Discussion

The current study aimed to investigate the level of NK, factors affecting knowledge and predictors of good NK in a sample of university students in the UK. The majority of participants were White, female, undergraduate students, younger than 25 years old, which is comparable to populations in similar studies^(4,8)^, suggesting that specific groups of students might be more interested in participating in health-related surveys. Most students had a normal BMI, and about one-third were overweight or obese (Table 1). These numbers are consistent with those found in a large cross-sectional study in the USA, where about one-third of students were overweight or obese^(8)^. In the UK, the study by Cooke & Papadaki^(4)^ found a slightly lower number of overweight and obese students (24 %). Although both studies used self-reported anthropometric measures, Cooke & Papadaki^(4)^ included a larger sample size (*n* 500) across thirty-seven universities in the UK (outside the London area), which might explain the differences found in the prevalence of obesity. In this study, the majority of students seemed to follow a healthy lifestyle in terms of not smoking and maintaining a healthy body weight even though only 24·7 % rated their health as ‘very good’ or ‘excellent’ (Table 1). Due to the specific characteristics of this population, it was expected that students would be more aware of healthy nutrition. Existing evidence suggests that adults with an increased education level in the UK have higher levels of NK compared to those with lower or no qualifications^(25)^. Also, about half of the participants (41·1 %) were from a healthcare field of study, such as Midwifery, Nursing, and Medicine, and were expected to have had some previous exposure on nutrition education during their courses, although the year of study was not included as an independent variable.

In the current study, students had a median NK score of 64 out of 88 (72·7 %) (Table 2). These scores were higher than the ones reported in studies previously undertaken in the UK (65·5 %)^(4)^ and Croatia (67·4 %)^(12)^ which used the same questionnaire (old version) to assess knowledge. The findings reported in the UK in 2013^(4)^ and the current study suggest that the level of knowledge of students in the UK has slightly improved over the last 5 years, although it is not clear to what extent the assessment method and characteristics of the sample population of the two studies (field of study and geographical differences) affected overall NK.

A closer investigation of individual responses showed that very few participants answered correctly the questions related to fat, salt and fibre, indicating gaps in knowledge about these nutrients and the foods containing them. This lack of knowledge could further explain the findings of previous studies reporting that university students consume high amounts of fats^(26,27)^ and salt^(28)^ and low amounts of dietary fibre^(27)^. A high number of students in this current study were aware of the healthiest meals and desserts from a list of options, which might be consistent with the fact that 64·2 % of students had a healthy BMI; however, the dietary habits and physical activity levels were not investigated in this study which means that valid conclusions cannot be made. Although the current study demonstrated an adequate level of knowledge in the section of diet–disease relationship and weight management questions, many students failed to answer correctly the questions about optimal practices to maintain a healthy body weight or prevent weight gain with some students answering that following a high protein diet, taking nutritional supplements or avoiding fat from diet are orthodox practices. Fad diets, which usually include the elimination of food groups from diet, are popular and common practices to lose weight, especially in females^(29)^ and overweight young adults^(30)^ due to their ‘promising’ quick and easy outcomes. Trying to lose weight is a concern occupying not only overweight, but also many students with a healthy body weight^(31)^. A study among 38 204 university students in the USA demonstrated that students with false perceived body weight were more likely to engage in unorthodox weight loss practices, and only one-third of those trying to lose weight did so by following a balanced diet and exercise^(31)^.

The current study found that less than half of the students (46·8 %) reached a ‘good’ level of NK (median score ≥ 64 points) (Table 2). Although participants studying a healthcare discipline had significantly higher median scores than students from non-healthcare disciplines (*P* < 0·05), ‘good’ level of NK was not positively associated with field of study (*P* = 0·555) or differed between students in healthcare and non-healthcare courses (*P* = 0·154). These findings are consistent with other studies demonstrating that students from Theoretical and non-Medical Practical Sciences had lower NK compared to students from Medical or other Health Sciences or those with a nutrition qualification^(17,20,21)^, justifying the initial speculation that students from a healthcare course had been somehow exposed to and were more knowledgeable about good nutritional practices. However, it is not clear whether this increased knowledge reaches a satisfactory level that could affect the dietary habits of students. Other studies have found that prior nutrition education or studying a health-related course did not significantly impact knowledge^(5,32)^, indicating that nutrition education interventions should be applied to students across all academic disciplines.

Age, ethnicity and perceived health were significant predictors of ‘good’ NK (Table 4). Students who rated their health as ‘very good’ or ‘excellent’ were more likely to have ‘good’ knowledge compared to students who rated having ‘poor’, ‘fair’ or ‘good’ health, implying that feeling healthier is related to better NK. These findings are in line with the study by Matthews *et al.*^(5)^ which found that students from Health Sciences felt more confident to claim that they have good knowledge of nutrition topics. Senior students were also found to have greater knowledge compared to junior students. This is consistent with the findings of similar studies^(18,19)^ and might partially explain the so-called phenomenon of ‘freshmen fifteen’, which refers to the belief that students gain fifteen pounds (6·8 kg) during their first year of studies^(33)^. Although pooled evidence from meta-analyses showed that the actual weight gain is much less (1·36 kg)^(34)^, body weight and dietary habits seem to start changing unfavourably during the first year of studies^(35,36)^, highlighting the importance of implementing interventions to increase knowledge and awareness of healthy eating in early academic years. The study also found that White students had higher levels of knowledge compared to students with Black, Asian or other/mixed ethnicity. This might be due to the different cultural and culinary traditions of the different ethnic groups. What is further alarming is that even dietetic students seem not to be knowledgeable of the food habits and health beliefs of individuals from different ethnic groups, as reported in McArthur *et al.*^(37)^. These findings suggest that the cultural background of students might play an important role on their dietary knowledge and behaviour, which may be overlooked in current health-promoting strategies.

Sample limitations of the current study include a non-stratified sample with a high dropout rate (37 %), as 190 out of 301 participants who entered the study and provided consent completed at least 90 % of the questions. BMI was calculated based on self-reported data, providing less accurate values (underreporting) when compared with data assessed with objective methods^(38)^. However, self-reported measures of BMI seem to have a small effect on associations observed in epidemiological studies and can still provide important information^(38)^. To the best of our knowledge, this is the first study that used the revised version of the GNKQ published in 2016, exploring the knowledge on updated nutritional information, such as oily fish, hidden sources of salt, alcohol intake, cooking methods, food labelling and optimal practices to maintain a healthy body weight. Besides Cooke & Papadaki^(4)^, who investigated NK as a predictor of food label use in a sample of university students in the UK, no similar studies have been conducted in the UK to investigate gaps in NK and factors affecting this knowledge in students. It should be noted though that the GNKQ-R included only multiple choice questions, which allowed students to guess the right answer or choose it by excluding the obvious wrong answer. Also, the relationship between knowledge and dietary habits as well as the impact of the environment and social support on dietary behaviour was not explored in this study. Students were recruited from two London-based universities, one of which provided only health-related academic disciplines. This resulted in having a high number of participants from healthcare courses which reduces the generalisability of our findings, although, concurrently, it provided the opportunity to explore the impact of the field of study on NK. It is important to note that both universities attract a high number of diverse students and international students, which is very common for London-based universities and might explain the differences in knowledge found between the different ethnic groups^(39)^. It may also imply that the lifestyle challenges and difficulties students face during the transition from high school to university might be more intense for the students who just relocated to the UK for studying. However, these are speculations and are not addressed by this research.

## Conclusions

Students demonstrated good knowledge in the section of healthy food choices; however, their knowledge about the nutrient sources of foods was inadequate. Gaps in knowledge were found regarding the intake of fats, salt and good practices of weight management, indicating areas for improvement when designing nutrition education interventions. When assessing knowledge, using a mixed-methods research design or enhancing the quantitative data with open-ended questions might help to elaborate and gain an in-depth understanding of students’ knowledge. When investigating NK, researchers should consider the academic discipline but also the different cultural and ethnic backgrounds of students, as this study found that White students and students from a healthcare field of study demonstrated higher levels of NK. Among students from a healthcare field of study, the majority did not manage to demonstrate a good level of NK, suggesting that nutritional education interventions would be beneficial to all students, irrespective of their course. Finally, more research is needed to investigate the reliability and validity of the sources of information that students use to gain knowledge on nutrition and weight management practices. In order to inform policy actions, future research needs to investigate to what extent NK affects students’ dietary habits, alongside the impact of the environmental and social factors on dietary behaviour.
